# Development of genomic instability-associated long non-coding RNA signature: A prognostic risk model of clear cell renal cell carcinoma

**DOI:** 10.3389/fonc.2022.1019011

**Published:** 2022-10-19

**Authors:** Dongfang Jiang, Tiange Wu, Naipeng Shi, Yong Shan, Jinfeng Wang, Hua Jiang, Yuqing Wu, Mengxue Wang, Jian Li, Hui Liu, Ming Chen

**Affiliations:** ^1^ Department of Urology, Danyang People’s Hospital, Danyang, China; ^2^ Department of Clinical Medicine, Medical School of Southeast University, Nanjing, China; ^3^ Department of Urology, Zhongda Hospital Affiliated to Southeast University, Nanjing, China; ^4^ Department of Urology, The Second People's Hospital of Taizhou, Taizhou, China; ^5^ Department of Urology, Yancheng Third People’s Hospital, Yancheng, China; ^6^ Department of Urology, Jinhu County People’s Hospital, Huaian, China; ^7^ Department of Urology, Binhai County People’s Hospital, Yancheng, China

**Keywords:** cell renal cell carcinoma, TCGA, long non-coding RNA, prognosis, bioinformatics

## Abstract

**Purpose:**

Renal clear cell carcinoma (ccRCC) is the most lethal of all pathological subtypes of renal cell carcinoma (RCC). Genomic instability was recently reported to be related to the occurrence and development of kidney cancer. The biological roles of long non-coding RNAs (lncRNAs) in tumorigenesis have been increasingly valued, and various lncRNAs were found to be oncogenes or cancer suppressors. Herein, we identified a novel genomic instability-associated lncRNA (GILncs) model for ccRCC patients to predict the overall survival (OS).

**Methods:**

The Cancer Genome Atlas (TCGA) database was utilized to obtain full transcriptome data, somatic mutation profiles, and clinical characteristics. The differentially expressed lncRNAs between the genome-unstable-like group (GU) and the genome-stable-like group (GS) were defined as GILncs, with |logFC| > 1 and an adjusted *p-value*< 0.05 for a false discovery rate. All samples were allocated into GU-like or GS-like types based on the expression of GILncs observed using hierarchical cluster analyses. A genomic instability-associated lncRNA signature (GILncSig) was constructed using parameters of the included lncRNAs. Quantitative real-time PCR analysis was used to detect the *in vitro* expression of the included lncRNAs. Validation of the risk model was performed by the log-rank test, time-dependent receiver operating characteristic (ROC) curves analysis, and multivariate Cox regression analysis.

**Results:**

Forty-six lncRNAs were identified as GILncs. LINC00460, AL139351.1, and AC156455.1 were employed for GILncSig calculation based on the results of Cox analysis. GILncSig was confirmed as an independent predictor for OS of ccRCC patients. Additionally, it presented a higher efficiency and accuracy than other RCC prognostic models reported before.

**Conclusion:**

GILncSig score was qualified as a critical indicator, independent of other clinical factors, for prognostic prediction of ccRCC patients.

## Background

Renal cell carcinoma (RCC) accounts for approximately 85% of urinary cancers generated from the kidneys, and the associated morbidity is growing continuously over recent years (1). Nearly 100,000 patients die from RCC annually all over the world, and more than 170,000 deaths were observed globally in 2018 according to recent statistics (2, 3). Histologically, RCC can be classified into five subtypes with unique characteristics, including clear cell (ccRCC), papillary (pRCC), chromophobe (cRCC), collecting duct (cdRCC), and unclassified RCC. About 70~80% of patients are diagnosed with ccRCC after tumor biopsy or nephrectomy (4). Generally, ccRCC with metastases is associated with high mortality, and over 25% of patients first diagnosed with ccRCC were reported to be distant metastatic with a 5-year survival rate of 0~10% (5). Besides, the number of deaths from ccRCC accounts for the most among all subtypes of kidney cancers.

Genomic instability has been widely acknowledged as a trigger to carcinogenesis and requires therapeutic intervention. Usually, cancer genomic instability promotes the development of carcinomatous characteristics (6, 7). The more frequently the genetic alterations arise, the more likely the genomic instability occurs during the cell cycle (8). A high level of genomic instability with numerous somatic mutations could lead to malignant progression, distant metastasis, and poor prognosis in multiple cancers (9–11). It was currently reported that genomic instability of various critical genes in RCC cells can affect metabolic features of the tumor and disturb the process of cell division, thus resulting in malignant progression (8, 12).

Long non-coding RNAs (lncRNAs) are defined as transcripts comprising over 200 nucleotides that are unable to encode proteins. They function as regulators equipped with diverse biological functions in tumor-associated signaling pathways, including epigenetic regulation, transcriptional regulation, and post-transcriptional regulation (13, 14). In multiple cancers, such as breast cancer, prostate cancer, colorectal cancer as well as RCC, aberrant lncRNAs were often detected at different processes of tumorigenesis. For example, dysregulated lipid-associated lncRNAs could be regarded as predictors of poor prognosis of cancer (15). Moreover, lncRNA-URRCC, which is overexpressed in RCC samples, was found to be related to poor prognosis and acceleration of cell proliferation, and invasion in the ccRCC (16).

Nowadays, although aberrantly expressed lncRNAs and genomic instability are both considered to play core roles in the carcinogenesis of renal cells, it is still unclear whether there is a clinical association between them (9–14). Notably, evidence unraveling the critical roles of lncRNAs in genomic instability maintenance and the prognostic significance of the genomic instability-associated lncRNAs in cancer patients remained to be identified. Therefore, in this study, we attempted to establish a risk-score model for predicting the prognosis of ccRCC patients based on statistics from the TCGA database.

## Methods

### Data retrieval and sample classification

We obtained full transcriptome data, somatic mutation profiles, and clinical characteristics of 539 patients from the TCGA database (https://cancergenome.nih.gov/). To identify the possible relations between lncRNAs and genomic instability, lncRNA expression profiles and somatic mutation profiles were analyzed. After the somatic mutation frequency calculation of every sample, all the samples were ranked in descending order. The top 25% and the bottom 25% of these samples were allocated to the genome-unstable-like group (GU) and the genome-stable-like group (GS), respectively. The differences in lncRNA expression levels between the two groups were evaluated with a significance analysis of microarrays (SAM). Differentially expressed lncRNAs with |logFC| > 1 and adjusted *p-value*< 0.05 in false discovery rate (FDR) were defined as genomic instability-associated lncRNAs (GILncs). According to expression levels of specific lncRNAs, all samples were separately assigned to GU-like type and GS-like type using hierarchical cluster analyses.

### Co-expression net and gene functional exploration

Co-expression regulatory net model was constructed to analyze the correlation between 46 lncRNAs and the corresponding susceptible mRNAs. The relevancy degrees were measured by Pearson correlation coefficients per cluster. Furthermore, enrichment analyses of most related mRNAs were conducted for function prediction, in terms of Gene Ontology (GO) terms and the Kyoto Encyclopedia of Genes and Genomes (KEGG) pathway (17). Statistics were processed and visualized with the ‘clusterProfiler’ R package (18).

### Quantitative real-time PCR analysis

RNA extraction kits (OMEGA, China) were used to extract RNA from the kidney tissue. The specific primers used were as follows: 5’ ACGCAGTGGATGAGAACGAA (LINC00460 forward) and 5’ GGGGTGACTTCAGAATGCGT (LINC00460 reverse); 5’CTTCACATTCTACACAGCCTCTCCT (AL139351.1 forward) and 5’GGTGTGGGTGAAGTAAAG AAAGC (AL139351.1 reverse); 5’ CTCACTGGAGCCG CCTAACTT (AC156455.1 forward) and 5’ CGTGTTGA GGACTACAGAAGAGGA (AC156455.1 reverse). mRNA expressions were normalized to GAPDH mRNA expression. Every experiment was repeated at least three times.

### Prognosis-related statistical analysis

To identify prognosis-related GILncs independently from other features of ccRCC patients, univariate and multivariate Cox proportional hazard regression analyses were performed. The qualified prognosis-related lncRNAs were considered for prognostic model construction, of which *p-values*< 0.05 was considered statistically significant.

Combining the expression of prognosis-related GILncs and coefficients from multivariate Cox regression, a genomic instability-associated lncRNA signature (GILncSig) for predicting prognosis was derived, and the computational formula for the same is as follows:


(1) $$\text{GILncSig (patient) = }\Sigma_{i=1}^{n}\text{coef }{\text{(lncRNA}}_{i}\text{) }*\text{ expr }{\text{(lncRNA}}_{i})$$

Where, GILncSig (patient) represents a prognostic risk score for ccRCC patients, lncRNA_i_ represents the i^th^ prognosis-related lncRNA, expr (lncRNA_i_) is the lncRNA_i_ expression level, and coef (lncRNA_i_) is the contribution of lncRNA_i_ to the risk score derived from multivariate Cox regression coefficients.

Classification between the low-risk group with low GILncSig and the high-risk group with GILncSig relying on the risk cutoff was computed by the median score of the patients in the training set. The accuracy of the predictive model for each group was evaluated by the Kaplan–Meier (KM) log-rank test, time-dependent receiver operating characteristic (ROC) curve analysis, and multivariate Cox regression analysis. KM survival curves were analyzed to determine correlations among all parameters, including clinical characteristics and GILncSig. Hazard ratios (HR), 95% confidence interval (CI), and *p-value* were standards for identifying independent prognostic indicators. ROC curves were utilized to evaluate the predictive effectiveness of the genome unstable lncRNA-based risk scores for the prognosis of ccRCC patients. A two-sided *p-value*< 0.05 threshold was considered statistically significant. All statistical analyses were conducted with R version 4.0.3.

## Results

### Sample cluster dependent on GILncs

**Figure 1:**
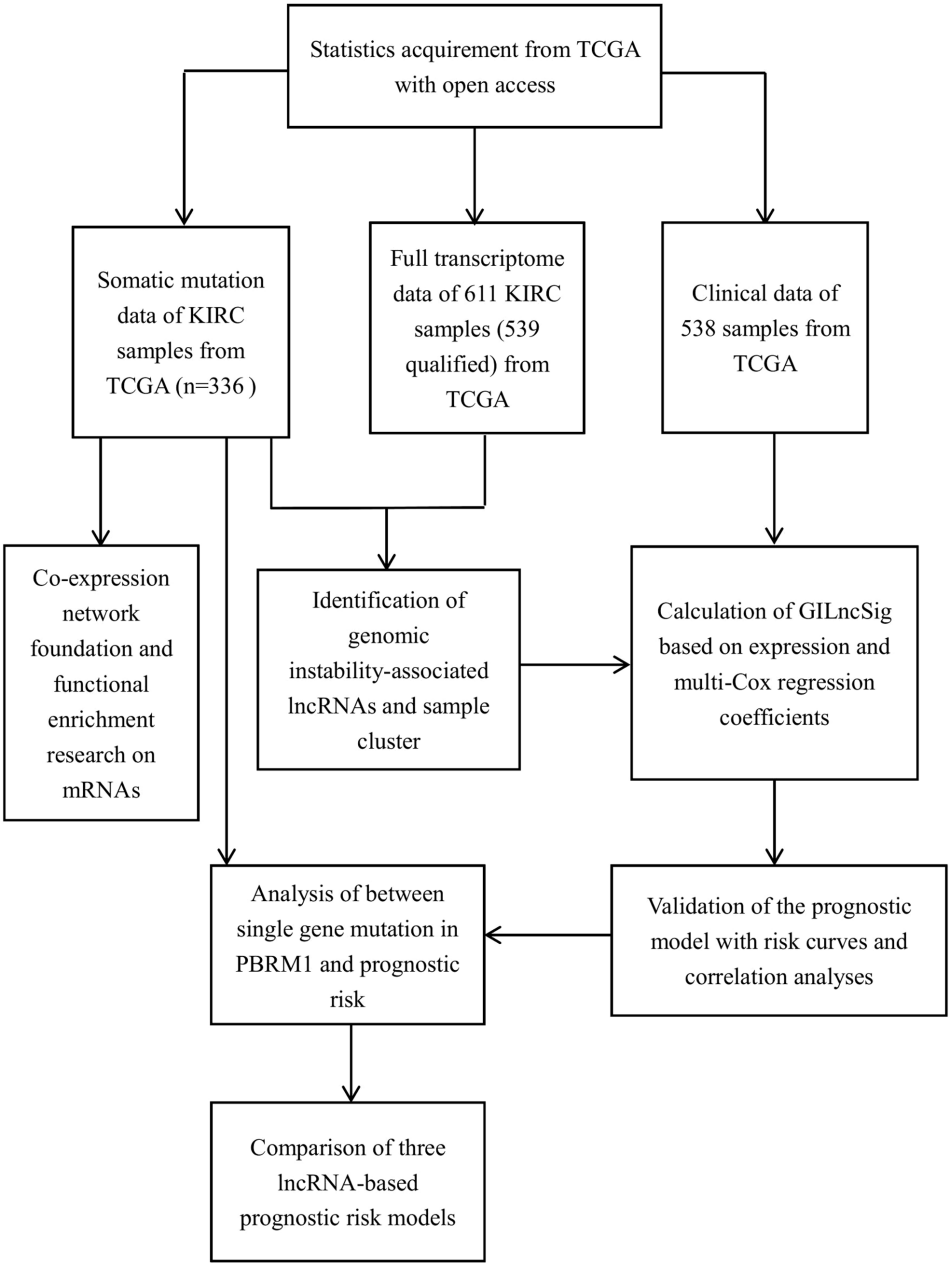
The flow chart of research processes.

**Figure 2:**
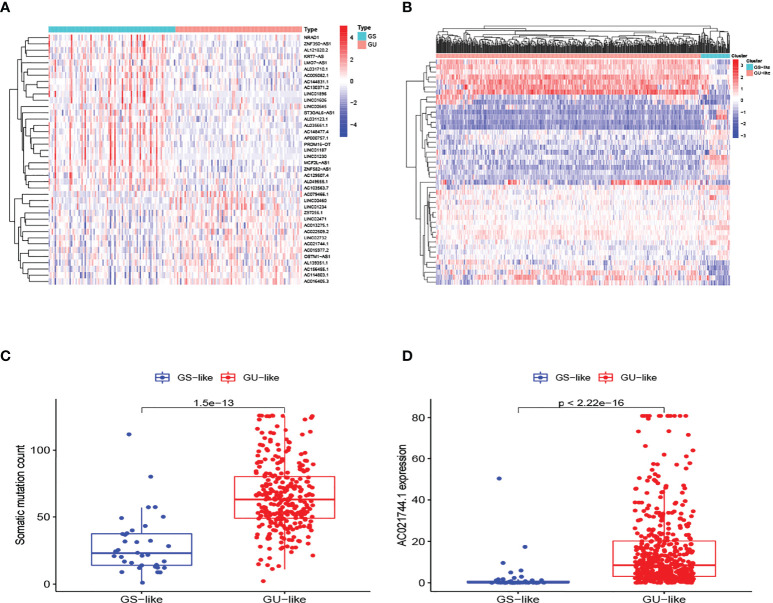
Identification of GILncs and whole sample cluster. **(A)** The heatmap plot of lncRNAs based on mutation frequency. **(B)** Hierarchical clustering analysis of all 539 samples. GU-like type is colored in red and GS-like type is colored in blue. **(C)** The boxplot of somatic mutation count comparison between GU-like type and GS-like type. **(D)** The boxplots of AC021744.1 expression level in the GU-like type and GS-like type.

The research process is shown in **Figure 1**. Detailed clinical characteristics of ccRCC patients including age, gender, grade, clinical stage, and TNM are described in **Table 1**, and no difference was detected between the subgroups. First, 170 samples were divided into the GU-like group and GS-like group, based on the cumulative number of somatic mutations. The expressions of 46 genes were significantly different between the two groups (**Figure 2A**). Using hierarchical cluster analyses, all samples were clustered based on the expression levels of qualified lncRNAs. Fifty-three and 486 samples were classified into GS-like type and GU-like type, respectively (**Figure 2B**). Consistently, the frequency of somatic mutations was significantly higher in GU-like type than in GS-like type (**Figure 2C**). For reverse verification, the expression of the novel GILnc AC021744.1 was compared between the two groups. As was expected, the expression of AC021744.1 was significantly higher in the GU-like type compared with the GS-like type (**Figure 2D**).

**Table 1:** Clinical information for three sets in ccRCC patients.

Covariates		Training set (n = 257)	Testing set (n = 256)	TCGA set (n = 513)	P-value
Age, n (%)	<65	164 (64.3)	176 (68.75)	340 (66.28)	0.276
	≥65	93 (36.19)	80 (31.25)	173 (33.72)	
Gender, n (%)	Female	90 (35.02)	86 (33.59)	176 (34.31)	0.805
	Male	167 (64.98)	170 (66.41)	337 (65.69)	
Grade	G1-2	111 (43.19)	120 (46.88)	231 (45.03)	0.5005
	G3-4	141 (54.86)	133 (51.95)	274 (53.41)	
	unknow	5 (1.95)	3 (1.17)	8 (1.56)	
Stage, n (%)	Stage I/II	95 (67.9)	91 (66.9)	311 (60.62)	0.501
	Stage III/IV	103 (40.08)	96 (37.50)	199 (38.79)	
	Unknow	2 (0.78)	1 (0.39)	3 (0.58)	
T stage, n (%)	T1/T2	160 (62.26)	169 (66.02)	329 (64.13)	0.4263
	T3/T4	97 (37.74)	87 (33.98)	184 (35.87)	
M stage, n (%)	M0	194 (75.49)	213 (83.20)	407 (79.34)	0.0339
	M1	48 (18.68)	30 (11.72)	78 (15.20)	
	Unknow	15 (5.84)	13 (5.08)	28 (5.46)	
N stage, n (%)	N0	112 (43.58)	117 (45.7)	229 (44.64)	1
	N1/N2	8 (3.11)	8 (3.12)	16 (3.12)	
	Unknow	137 (53.31)	131 (51.17)	268 (52.24)	

### LncRNA-mRNA co-expression net and biological function prediction

**Figure 3:**
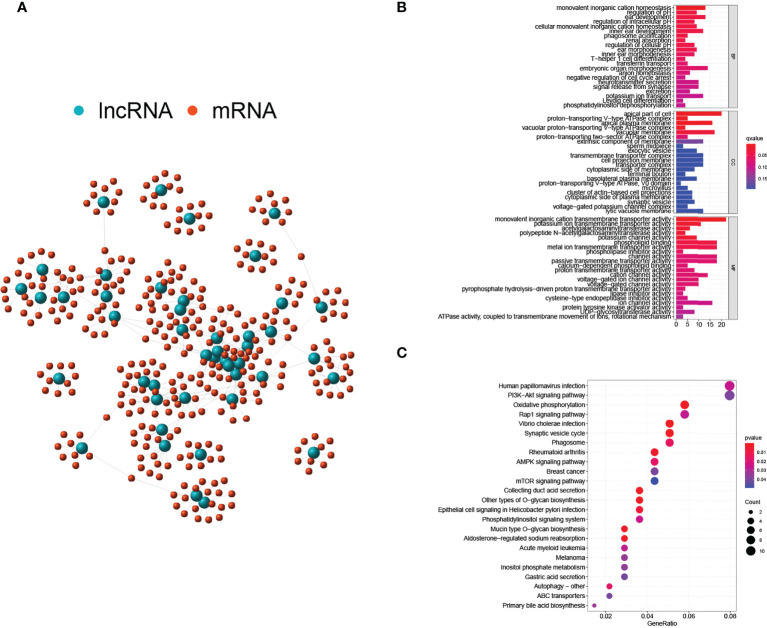
Regulatory network and functional analyses. **(A)** Co-expression network of GILnc and targeted mRNAs. **(B)** Bar plots of GO terms enrichment analysis. **(C)** The bubble plot of KEGG pathway enrichment analysis.

Interactions between lnRNAs and mRNAs were visualized in a network consisting of nodes and lines (**Figure 3A**). Green nodes represented critical GILncs, and red nodes represented the 10 most related mRNAs regulated by lncRNAs. The lines connecting them showed the degree of relevancy. To further predict the potential biological roles of mRNAs in ccRCC, the functional enrichment analysis was employed for GO terms and KEGG pathways research. As is displayed in **Figure 3B**, the ‘monovalent inorganic cation homeostasis’ (GO:0055067, *p*-*value* = 1.58×10^-6^) and ‘ear development’ (GO:0043583, *p*-*value* = 6.98×10^-5^) were GO terms of biological process (BP) that most genes are involved in. In terms of cellular component (CC), the number of genes that enriched in the ‘apical part of cell’ (GO:0045177, *p*-*value* = 5.70×10^-6^) was the greatest, reaching 20. As far as molecular function (MF) was concerned, most genes were likely to participate in the ‘monovalent inorganic cation transmembrane transporter activity’ (GO:0016324, *p*-*value* = 0.0001). Further, KEGG enrichment analysis was performed to find the biological signal pathways in which GILncs might be involved. In accordance with the bubble diagram (**Figure 3C**), included genes were mostly inclined to concentrate on KEGG pathways of ‘Human papillomavirus infection’ (n = 11, *p*-*value* = 0.026) and ‘PI3K-Akt signaling pathway’ (n = 11, *p*-*value* = 0.039).

### Establishment of GILncSig and prognostic risk model

Using multivariate Cox regression analysis, 3 lncRNAs namely LINC00460, AL139351.1, and AC156455.1, that were closely related to OS were selected for the model establishment. To validate the result, human renal cells were used to confirm the high expression of the included lncRNAs.

**Figure 4:**
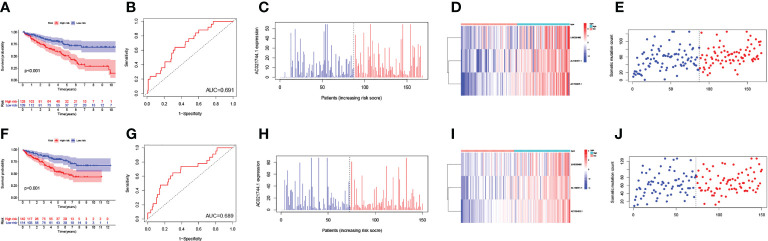
Validation of the GILncSig model. **(A)** The survival analysis in training set; **(B)** The ROC curve in training set; **(C)** The expplot analysis in training set; **(D)** The heatmap in training set; **(E)** The mutplot analysis in training set; **(F)** The survival analysis in testing set; **(G)** The ROC curve in testing set; **(H)** The expplot analysis in testing set; **(I)** The heatmap in testing set; **(J)** The mutplot analysis in testing set.

Next, all ccRCC samples were randomly allocated to the training set (n = 257) and the testing set (n = 256) for further validation of the prognostic risk model. The GILncSig was utilized as the index for risk group classification. To figure out the association between risk score and prognosis, survival analyses of both the training set and testing set were performed. KM curves of OS were drafted to compare the survival outcomes (**Figures 4A**, **F**) in the two sets, and both showed significantly better OS in the low-risk group than in the high-risk group (*p-value*< 0.001). Areas under the curve (AUC) values of ROC curves were determined to assess the reliability of our model (**Figures 4B**, **G**). AUC values of the training set (AUC = 0.691) and the testing set (AUC = 0.689) were simultaneously close to 0.7, indicating relatively high effectiveness for prognosis prediction. The correlation between prognostic risk score and AC021744.1 expression was illustrated in expplots (**Figures 4C**, **H**) and heatmaps (**Figures 4D**, **I**). The results suggested that as the risk score increases, the expression level of lncRNA AC021744.1 would enhance consistently in both the training set and the testing set. In addition, lncRNA LINC00460, AL139351.1, and AC156455.1 were found to be upregulated when the risk score was higher, which further clarified these three lncRNAs as high-risk genes. The mutplots (**Figures 4E, J**) showed that for both the training and testing set, higher somatic mutation counts were observed for higher risk scores.

### Relevance between risk score and somatic mutation count/AC021744.1 expression

**Figure 5:**
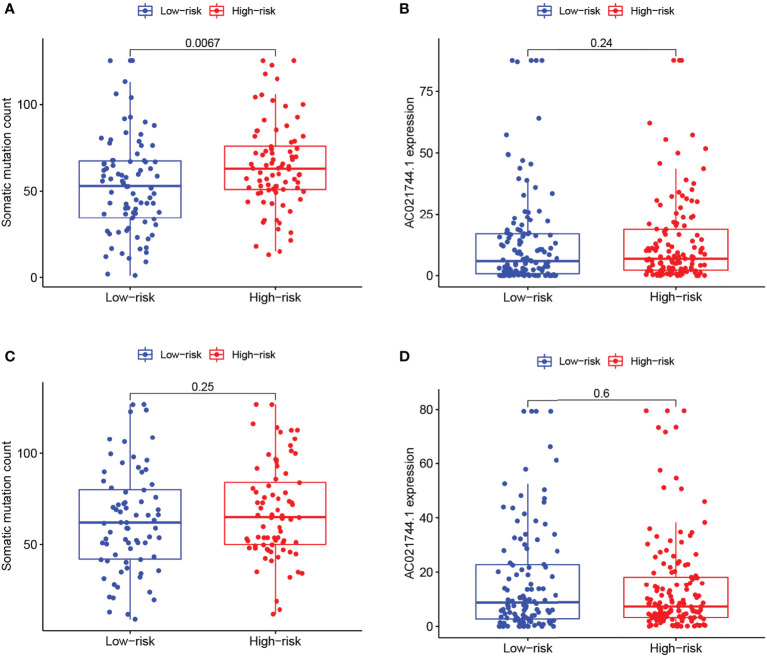
Risk correlation analysis. **(A, C)** Boxplots of correlation between risk levels and somatic mutation count in training group and testing group. **(B, D)** Boxplots of correlation between risk levels and AC021744.1 expression in training group and testing group.

As demonstrated in the two sets of box plots in **Figure 5**, the association between prognostic risk score and mutation counts or AC021744.1 expression level was further assessed in pairs. A significantly positive trend could be illustrated between mutation frequency and risk of prognosis in the training set (*p-value* = 0.0067, **Figure 5A**), while no significant association was detected in the testing set (**Figure 5C**). No association between risk score and the AC021744.1 expression level was found in the training set, nor in the testing set (**Figures 5B**, **D**).

### Validation of GILncSig as an independent prognostic factor from clinical characteristics

To further investigate whether GILncSig could be identified as an independent predictor of OS, several potential prognostic indicators were comprehensively researched with the integration of univariate and multivariate Cox regression analysis. Factors of interest included age, gender, pathological grade, clinical stage, and GILncSig (**Table 2**). The Cox regression analysis revealed that the GILncSig Score could further be identified as an independent prognostic factor affecting OS from other potential factors in the testing set (HR = 1.110, 95% CI 0.987-1.248, *p-value* = 0.019) and TCGA set (HR = 1.015, 95% CI 1.007-1.023, *p-value*< 0.000). Similarly, pathological grade showed a significant association with survival outcomes in the testing set (HR = 1.633, 95% CI 1.146-2.327, *p-value* = 0.007) and TCGA set (HR = 1.428, 95% CI 1.131-1.802, *p-value* = 0.003), as well. Meanwhile, age and the clinical stage could significantly influence the survival of ccRCC patients in all sets.

**Table 2:** Univariate and Multivariate Cox regression analysis of the GILncSig and OS in different sets.

Variables		Univariable model			Multivariable model		
		HR	95% CI	P-value	HR	95% CI	P-value
Training set (n =257)							
GILncSig	High/Low	1.271	1.148-1.407	0.000	1.110	0.987-1.248	0.081
Age		1.027	1.008-1.046	0.595	1.025	1.004-1.046	0.019
Gender	Female/Male	0.889	0.577-1.371	0.000			
Grade	(3-4)/(1-2)	2.024	1.527-2.683	0.000	1.268	0.929-1.731	0.135
Stage	(III/IV)/(I/II)	1.845	1.546-2.203	0.000	1.656	1.353-2.026	0.000
Testing set (n = 256)							
GILncSig	High/Low	1.098	1.060-1.136	0.000	1.044	1.004-1.086	0.030
Age		1.030	1.010-1.049	0.003	1.035	1.014-1.057	0.001
Gender	Female/Male	1.039	0.652-1.656	0.872			
Grade	(3-4)/(1-2)	2.538	1.867-3.449	0.000	1.633	1.146-2.327	0.007
Stage	(III/IV)/(I/II)	1.948	1.581-2.401	0.000	1.692	1.329-2.154	0.000
TCGA set (n = 513)							
GILncSig	High/Low	1.112	1.079-1.146	0.000	1.015	1.007-1.023	0.000
Age		1.029	1.015-1.042	0.000	1.031	1.016-1.046	0.000
Gender	Female/Male	0.964	0.703-1.323	0.821			
Grade	(3-4)/(1-2)	2.268	1.845-2.787	0.000	1.428	1.131-1.802	0.003
Stage	(III/IV)/(I/II)	1.896	1.658-2.168	0.000	1.664	1.428-1.938	0.000

**Figure 6:**
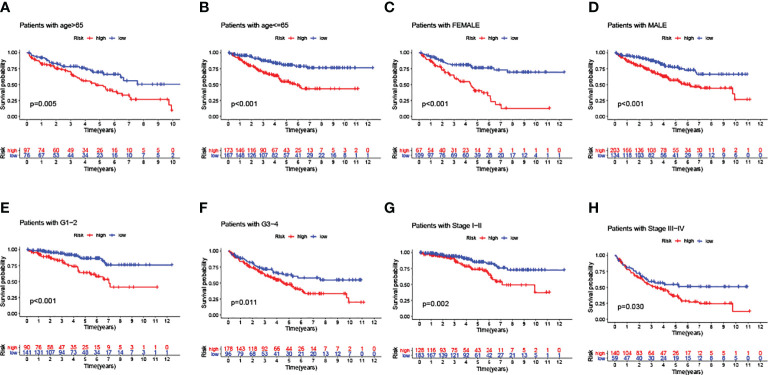
Predictive consistency analyses of GILncSig in populations with different clinical characteristics. **(A, B)** KM curves: Comparisons of OS between ccRCC patients with high and low risk, in old and young groups. **(C, D)** KM curves: Comparisons of OS between ccRCC patients with high and low risk, in male and female groups. **(E, F)** KM curves: Comparisons of OS between ccRCC patients with high and low risk, in pathologically early and advanced groups. **(G, H)** KM curves: Comparisons of OS between ccRCC patients with high and low risk, in clinically early and advanced groups.

In addition, a series of KM log-rank analyses were performed to further validate GILncSig as an independent prognostic factor in different subgroup samples. In each pair of subgroups, survival time was positively correlated with a risk score, under the effects of age (**Figures 6A**, **B**), gender (**Figures 6C**, **D**), pathological grade (**Figures 6E**, **F**), and clinical stage (**Figures 6G**, **H**).

To conclude the results above, the GILncSig score could be deemed as a prognostic predictor with consistent independence, which was negatively correlated with the OS of ccRCC patients.

### Relationship between risk and oncogene mutation

**Figure 7:**
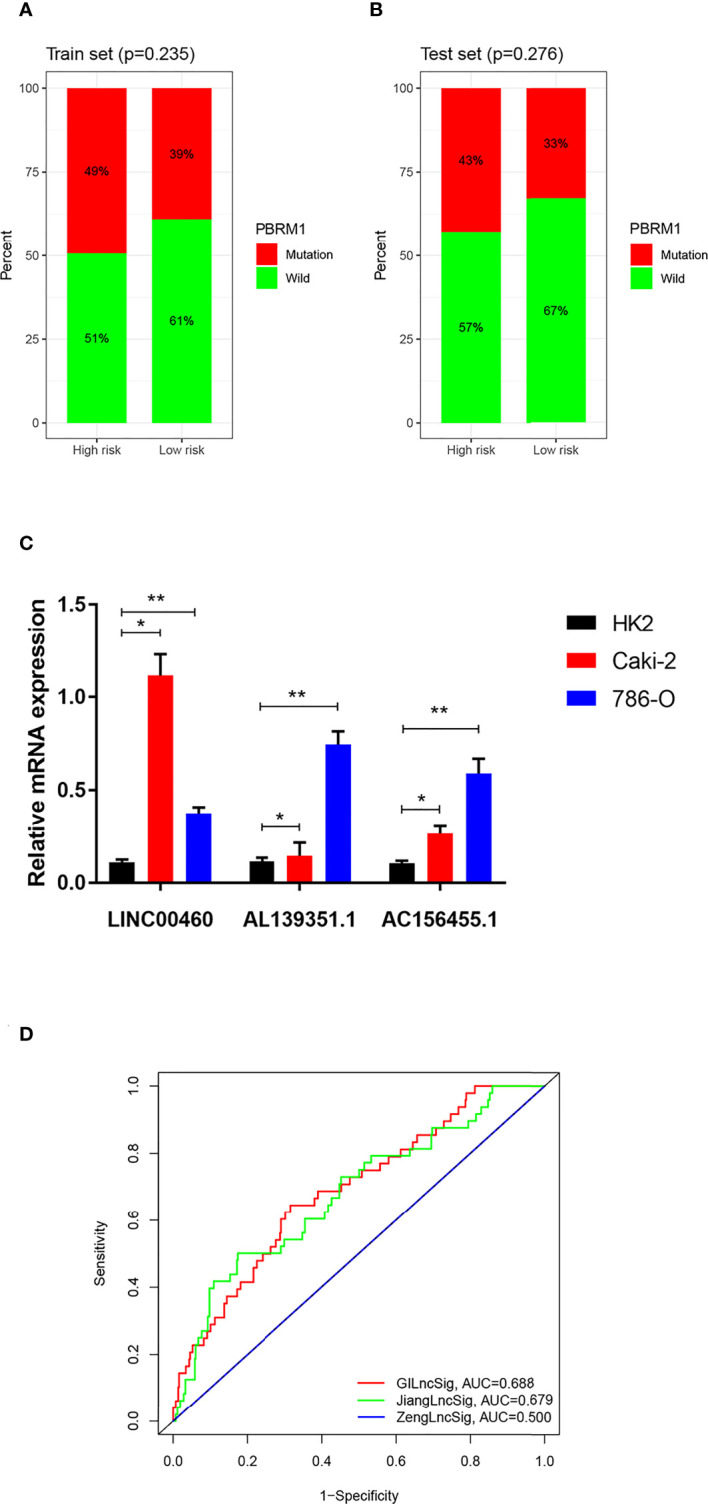
**(A, B)** Carcinogenic function analysis of GILncSig based on single geng mutation of PBRM1: Proportional bar plots of mutation type and wild type of PBRM1 in samples of training set and testing set. **(C)** mRNA expression of LINC00460, AL139351.1 and AC156455.1 in HK2, 786-O and Caki-2 cells according to qRT-PCR analysis. **(D)** Comparison among three lncRNA-based prognostic models based on AUCs: ROCs of GILncSig, SunLncSig and ZengLncSig. * means p<0.05, and ** means p<0.01.

PBRM1 has been recognized as a classic oncogene taking part in genomic instability and a high mutation frequency in PBRM1 was related to the occurrence of ccRCC (19). To figure out whether the new signature of risk estimation in ccRCC can predict prognosis, the single genetic mutation count was calculated in the training and testing sets. Although the proportion of mutation appeared to be larger in the high-risk group in both the two sets (**Figures 7A**, **B**), no statistical significance was probed (training set *p-value* = 0.235, testing set *p-value* = 0.276).

### Expression level of GILnc in different cell lines

As shown in **Figure 7C**, the expressions of LINC00460, AL139351.1, and AC156455.1 determined by the qPCR analysis were higher in RCC cells 786-O and Caki-2 as compared with the normal renal cells.

### Comparison of lncRNA-Related prognostic prediction models

To evaluate the efficiency of our prognostic model of GILncSig, it was compared with the other two published lncRNA-related prognostic prediction models for ccRCC. AUCs of the three ROC curves corresponding to the three models represented relative predictive accuracy. As illustrated in **Figure 7D**, the AUC of ZengLncSig (20), a six-lncRNA-based risk model, was 0.500, while that for another five immune-related lncRNA signatures of Sun (21), was 0.679. Accordingly, our GILncSig of 3 lncRNAs with the AUC of 0.688 exhibited the most effective prediction of prognosis for patients with ccRCC.

## Discussion

ccRCC is the most prevalent histological subtype of kidney cancer, contributing to a major part of yearly mortality related to cancers (4, 5). While lncRNAs have become increasingly recognized for their multiple biological roles in the tumorigenesis process in ccRCC, studies on their role in prognostic risk prediction are still insufficient (22–24). The occurrence of somatic mutation in cancer-related genes plays a critical role in the induction of RCC, and there is an increasing number of etiological studies on genomic instability. For instance, Wang W et al. reported in 2012 that the genomic instability existing in the DNA repair gene Ku70 contributed to causing RCC (25). Moreover, genomic DNA hypomethylation, which promoted the genomic instability of the global genome was proved as a hallmark of RCC risk by Mendoza-Pérez J et al., offering further hypotheses on the etiology of the RCC tumorigenesis (26). In 2019, Renzo G DiNatale et al. demonstrated that the mutation on TCEB1 could diminish the suppressive effects of the Von Hippel-Lindau (VHL) gene in ccRCC. Therefore, molecular events contributing to high genomic instability were proved to enforce aggressiveness and adverse clinical outcomes in ccRCC patients (27–29). In recent years, several studies investigated the association between survival outcomes and different clusters of lncRNAs in ccRCC samples and established risk models to validate the predictive ability of lncRNAs. In an earlier study by Sun Z et al., an immune-related signature, synthesized from 5 lncRNAs (AC008105.3, LINC02084, AC243960.1, AC093278.2, and AC108449.2) extracted from the TCGA database, could successfully predict the clinical outcomes (21). Zeng JH et al. proposed a practical six lncRNA-based prognostic risk model (CTA−384D8.35, CTD−2263F21.1, LINC01510, RP11−352G9.1, RP11−395B7.2, and RP11−426C22.4) based on the expression levels of involved non-coding genes in ccRCC samples (20). However, prognostic models with the theme of GILncs are rarely reported. Therefore, when constructing predictive models for prognostic risk of ccRCC patients, we were encouraged to derive an original index, GILncSig score, and carry out a comprehensive analysis to validate relations among GILncs and clinical outcomes (30–32).

We eventually screened out 46 GILncs equipped with differential mutation frequency in 539 samples from the TCGA database with outright open access. After seriatim referring to correlative literature, it was observed that very few studies discussed non-coding genes AC016405.3, AC114803.1, AC156455.1, AL139351.1, OSTM1-AS1, and AC015977.2 in GILncSig computation. Nevertheless, the highly mutant lncRNA AC021744.1 was reported to be an indicator of poor prognosis among patients with hepatocellular carcinoma (HCC). The overexpression of AC021744.1 could directly lead to shorter recurrence-free survival (RFS) time because of severe liver fibrosis (33). As the only known oncogene relevant to genomic instability in our model, AC021744.1 was selected for the current study. In accordance with the individual distribution of 46 genome-unstable lncRNAs, all 539 samples were clustered as GS-like type (n = 53) and GU-like type (n = 486). In addition, Bao S reported in 2019 (34) that AC021744.1 was a gene instability-related lncRNA significantly correlated with the gene instability-driving gene UBQLN4 and played an important role in the occurrence and development of breast cancer. Hence, AC021744.1 was also considered a representative genomic instability-associated lncRNA for further validation. It was observed that with the presence or absence of genomic instability, levels of somatic mutation count would appear low and high, respectively, thereby verifying the positive association among the included parameters.

The detailed construction of the co-expression network among genomic instability-related lncRNAs and regulated mRNAs included quantitative synthesis and analysis of the relevance between lncRNAs and mRNAs. Unfortunately, further co-regulation predictions from any other databases were not obtained to add to the predictions of co-expression among genes and the lncRNAs of interest. We assumed it was because the functions and signaling pathways of genomic instability-associated lncRNAs were rarely studied or reported in the literature. Due to these limitations, more original sequencing statistics are anticipated for further co-expression or ceRNA analyses.

In the meantime, GO and KEGG enrichment analyses of susceptible mRNAs were conducted for biological function forecast. Noticeably, mRNAs targeted downstream were highly enriched at the sites of the ‘PI3K-Akt signaling pathway’, in line with the result of KEGG analysis. PI3K/Akt signaling pathway has been mentioned in numerous studies on carcinomas and is reported to participate in the malignant progression and poor prognosis of various cancers, including RCC (35). Hence, the impacts of genomic instability-related lncRNAs on the prognosis of ccRCC patients could be viewed from another aspect. When the upregulated and downregulated gene was analyzed separately in GO analyses, it was found that among the 46 genomic instability-associated lncRNAs, most upregulated lncRNAs significantly enriched GO terms while the enrichments due to a few downregulated lncRNAs candidates were insignificant. Therefore, we synthesized the enrichment to summarize the enrichment of GILncs on GO terms, no matter whether it was significant or not (**Figure 3B**).

After the establishment of the GILncSig model, samples could be accordingly divided into a high-risk group and a low-risk group. Evidently, the somatic mutation frequency and expression of high-risk genes, LINC00460, AL139351.1, and AC156455.1, were enhanced when the prognostic risk was higher. Meanwhile, ccRCC manifested more malignant attributes leading to poorer OS concluded by the incremental expression level of the cancer-associated gene, AC021744.1. As no biological functions of AL139351.1 and AC156455.1 have been found, attention should be paid to LINC00460, a dysregulated lncRNA reported in RCC in 2018 (36). It has been validated that LINC00460 functions as a competing endogenous RNA (ceRNA) in co-expression and promotes the malignant development of multiple cancers, including prostate cancer (37), skin cancer (38), hepatocellular cancer (39), colorectal cancer (40) and so on, except for RCC. Coincidentally, Zhang D et al. researched LINC00460 as well and when identifying a three-lncRNA prognostic signature (41), they speculated LINC00460’s competing endogenous feature for its overexpression in ccRCC but did not mention its potential genomic instability. Controversially, the mutation frequency and expression were not found to be consistently differential when quantitative analyses were performed (**Figure 5**), probably due to the uncertain carcinogenic mechanism in ccRCC. In addition, our GILncSig Score model possessed great independence as a prognostic predictor from other significant clinical factors, such as age, gender, pathological grade, and clinical stage, showing its compatibility for all kinds of ccRCC patients with different clinical characteristics. Besides, when comparing our model with former lncRNA-related signatures, it is worth noting that our GILncSig model showed better prognosis efficiency with a higher AUC of 0.688 (**Figure 7C**).

It is widely acknowledged that RCC is insensitive to radiotherapy and chemotherapy, and hence, remedies targeted to specific genes or immune checkpoint inhibitors (ICIs) are now being explored (42, 43). Since Braun DA et al. (43) clinically validated the alternations of PBRM1 as a biomarker of ICI response in RCC in 2019, and Carril-Ajuria L et al. (19) demonstrated the prognostic and predictive value of PBRM1 in ccRCC in the same year, more and more researchers are exploring relevant genes, which could be hallmarks of immune targeted therapy of PBRM1-mutant ccRCC. For example, in the recent 2021 conference of European Urology, Hagiwara M et al. (44) proposed that poly ADP-ribose polymerase-1 (PARP1) could be a marker of the efficacy of immunotherapy for patients with PBRM1-mutant ccRCC, the higher expression of which suggested poorer prognosis and higher drug resistance. In our study, 46 differentially expressed GILncs between genome-unstable and genome-stable-like groups were found, among which LINC00460, AL139351.1, and AC156455.1 were validated as significant independent prognostic factors and considered for the construction of the risk model. Furthermore, a partially positive correlation was observed between the risk score and the mutation of PBRM1 in the training and the testing set. The mutation of PBRM1 was more likely to appear in the high-risk group for both the training and testing sets, while no statistical significance was discovered. Though the difference was insignificant, this still led us to the potential of the risk model to predict response to ICI treatment for patients with PBRM1-mutated ccRCC. We assumed that it owed to the limits of sample size, and it remains to be confirmed in the future under the condition of a larger sample size or experiments *in vivo/vitro.*

However, some limitations in the current study could not be neglected. Owing to the lack of other independent cohorts to perform validation, the same datasets were applied to set the training group and test group for internal validation. Admittedly, by changing the distribution of samples, the distribution density of repeated samples would increase, and thus we anticipate more data from independent cohorts for further validation.

In addition, due to the lack of an extra independent dataset for external validation, the qPCR analysis comparing the mRNA expression in ccRCC cell lines, and the normal renal tubular epithelial cells was carried out, showing that GILncs of the risk model were expressed significantly higher in the tumor cell lines. As the three prognostic GILncs were overexpressed in ccRCC, we did perform clone formation and Transwell experiments to explore the effects of these lncRNAs on proliferation, migration and invasion of RCC cells at the beginning. However, no significant differences were observed in cells’ proliferation, migration and invasion compared to controls, after knocking down the three prognosis-associated GILncs in either Caki-2 or 786-o cell lines, respectively. Therefore, we hypothesized that these GILncs did not affect the prognosis of patients by directly promoting the aggressiveness of RCC cells and further experimental exploration are expected.

Finally, since the data were extracted retrospectively from databases, the outcomes may be not accurate enough, and prospective validations based on experimental results would make the results more persuasive. For example, lncRNA AC021744.1 was identified as an oncogene related to the poor prognosis of HCC patients (33), but its carcinogenesis in RCC still lacks further proof from experimental studies. Consequently, the arguments using AC021744.1 could not completely support the correlation between the risk score and OS. Further, functions of AL139351.1 and AC156455.1 included in GILncSig remain to be discovered.

To sum up, the GILncSig Score, an original index calculated with the coefficients and expression levels of GILncs, is qualified to be a critical indicator, independent of other clinical factors, for predicting the prognostic risk of ccRCC patients.

## Data availability statement

Publicly available datasets were analyzed in this study. This data can be found here: TCGA Database ID: TCGA-KIRC.

## Author contributions

JL, HL, and MC designed the study; DJ, TW, NS, and HJ conducted the study and maintained the data; YW analyzed the data and made the figures; MW helped draft the paper; JW and YS helped correct a major of the grammatical mistakes and made a great contribution to this revision. All authors contributed to the article and approved the submitted version.

## Funding

Medical Research Foundation of Jiangsu Province (Z2019024).

## Acknowledgments

We thank Bullet Edits Limited for linguistic editing and proofreading of the manuscript.

## Conflict of interest

The authors declare that the research was conducted in the absence of any commercial or financial relationships that could be construed as a potential conflict of interest.

## Publisher’s note

All claims expressed in this article are solely those of the authors and do not necessarily represent those of their affiliated organizations, or those of the publisher, the editors and the reviewers. Any product that may be evaluated in this article, or claim that may be made by its manufacturer, is not guaranteed or endorsed by the publisher.
